# Development and Performance Evaluation of Wearable Respiratory Self-Training System Using Patch Type Magnetic Sensor

**DOI:** 10.3389/fonc.2021.680147

**Published:** 2021-08-03

**Authors:** Hyo Kyeong Kang, Hojin Kim, Chae-Seon Hong, Jihun Kim, Jin Sung Kim, Dong Wook Kim

**Affiliations:** ^1^Department of Integrative Medicine, Yonsei University College of Medicine, Seoul, South Korea; ^2^Department of Radiation Oncology, Yonsei Cancer Center, Yonsei University College of Medicine, Seoul, South Korea

**Keywords:** radiation therapy, respiratory monitoring, respiratory self-training, patch-type magnetic sensor, micro-electro-mechanical-system (MEMS)

## Abstract

**Purpose:**

Respiratory training system that can be used by patients themselves was developed with a micro-electro-mechanical-system (MEMS)-based patch-type magnetic sensor. We conducted a basic function test and clinical usability evaluation to determine the system’s clinical applicability.

**Methods:**

The system is designed with a sensor attached to the patient’s chest and a magnet on the back to monitor the patient’s respiration by measuring changes in magnetic intensity related to respiratory movements of the thoracic surface. The system comprises a MEMS-based patch-type magnetic sensor capable of wireless communication and being applied to measurement magnets and mobile applications. System performance was evaluated by the level of systemic noise, the precision of the sensor in various breathing patterns, how measurement signals change for varying distances, or the presence or absence of material between the sensor and the magnet. Various breathing patterns were created using the QUASAR respiratory motion phantom; the data obtained were analyzed using the fitting and peak value analysis methods.

**Results:**

The sensor had a noise ratio of <0.54% of the signal; the average errors in signal amplitude and period for breathing patterns were 78.87 um and 72 ms, respectively. The signal could be measured consistently when the sensor–magnet distance was 10–25 cm. The signal difference was 1.89% for the presence or absence of a material, indicating that its influence on the measurement signal is relatively small.

**Conclusion:**

The potential of our MEMS-based patch-type wearable respiratory self-training system was confirmed *via* basic function tests and clinical usability evaluations. We believe that the training system could provide thorough respiratory training for patients after a clinical trial with actual patients confirming its clinical efficacy and usability.

## Introduction

The goal of radiation therapy is to irradiate a tumor with a prescribed dose to treat cancer while delivering the minimum required radiation dose to the nearby normal tissues to minimize side effects. To achieve this goal, efforts have been made by advancing radiation therapy equipment and treatment methods, including three-dimensional conformal radiation therapy (3D-CRT), intensity-modulated radiation therapy (IMRT), volumetric modulated arc therapy (VMAT), and stereotactic body radiation therapy (SBRT). Additionally, accurate localization of the tumor and surrounding organ at risks (OARs) is required for carrying out an effective radiation therapy. The locations of tumors and organs in the thorax or abdomen tend to change over time during breathing, so the consideration of related movements during the treatment is essential. According to the American association of physicists in medicine (AAPM) task group (TG) Report 76, the displacement of lung tumor in lower lobe could be a maximum of 18.5 mm, and the diaphragm, which is one of the abdominal organs, moved up to 101 mm during deep breathing. Regarding the tumor’s or OAR’s internal motion due to respiration, the international commission on radiation units and measurements (ICRU) Report 62 recommends radiation therapy to use the internal target volume (ITV), the clinical target volume (CTV) added with the inner margin (1). However, delivering the prescribed dose to the extended target volume while considering internal motion may increase the chance of the radiation induced complications on OARs (2–4).

Various studies have been conducted on methods for managing the respiration to reduce the uncertainty of radiation therapy caused by the motion of the target and adjacent OARs (5–7). The management methods of respiratory motions are the motion-encompassing methods, respiratory gating methods, breath-hold methods, forced shallow breathing with abdominal compression methods, and real-time tumor-tracking methods (8). Appropriate respiratory motion monitoring should be accompanied for the respiratory gating methods, breath-hold methods, and real-time tumor-tracking methods. Abdominal surface motion can be tracked either by using an artificial marker placed on the patient’s abdomen through which surface images can be captured using a secured camera and analyzed without using a marker, or a marker can be implanted in patient’s body, and its motion can be tracked in real-time. According to Philippe Giraud et al., radiotherapy accompanied by respiratory management reduced dosimetric parameters that predict cardiopulmonary and esophageal toxicities (9).

For efficient respiratory motion management, regulating patient’s breathing is essential, and previous studies have reported the usefulness of respiratory training for this purpose (5, 10, 11). Venkat et al. reported that respiratory training using an audiovisual biofeedback device resulted in a >50% reduction in displacement variation between breathing cycles and a >70% reduction period variation compared to free breathing (10). Respiratory training is recommended before treatment to maintain constant breathing patterns in patients whose respiratory motion should be considered for radiotherapy. The following elements are necessary for this goal: i) time other than radiotherapy time for respiratory training, ii) space for training and training equipment, and iii) workforce for helping with the training and monitoring the patient. Thus, providing respiratory training to all patients can be demanding the large effort and resource for medical institutions.

Further to previous work, which reported on the basic performance of a respiratory training system based on a micro-electro-mechanical-system (MEMS) sensor (12), we upgraded the system with a small patch-type sensor capable of wireless communication by having Bluetooth module introduced to MEMS sensor. In addition, a respiration signal is displayed on the user’s smartphone, and a mobile application was developed for respiration training, so a system was created to allow patients to do training anywhere, anytime, with ease. In this study, we evaluated the performance of the sensor used in our in-house respiratory monitoring and training system, and confirmed the system’s clinical usability.

## Materials and Methods

### Settings for Sensor Evaluation

**Figure 1:**
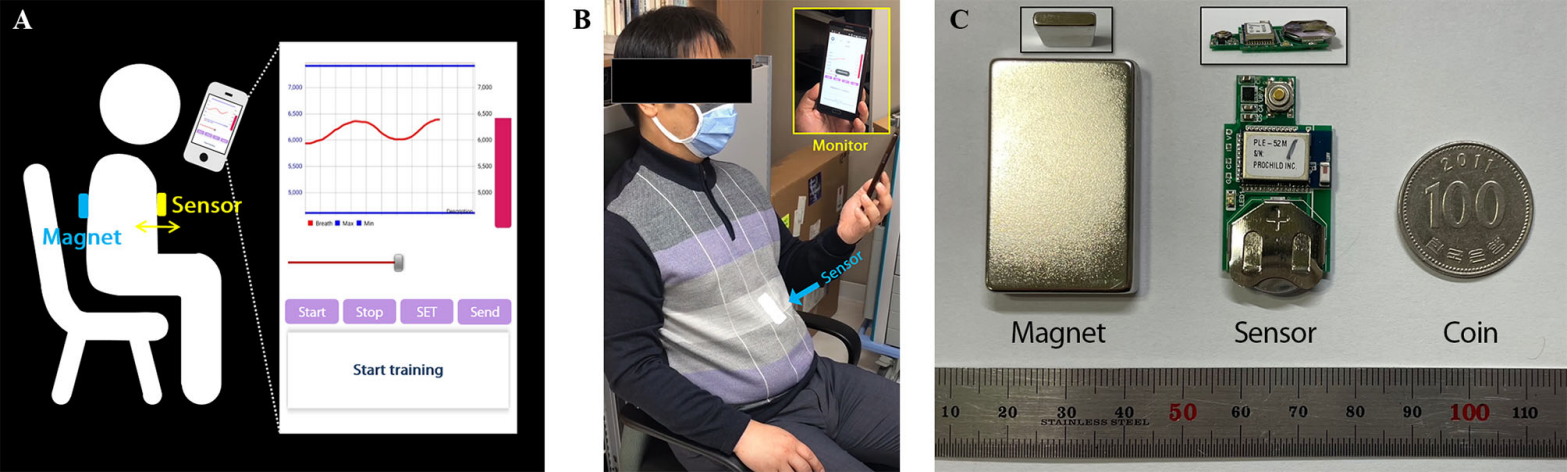
Overview of the respiratory monitoring and training system: **(A)** Illustration of the system overview. **(B)** Setup for sitting position. **(C)** The components of the system; Sensor and magnet are compared with coin to show their size.

As shown in **Figures 1A, B**, the system is designed to monitor respirations by attaching the sensor to patient’s chest and placing a magnet on the back. The chest surface movements according to respiration are displayed by variation of magnetic field intensity, taking advantage of the property of varying strengths of a magnetic field for change in the distance between sensor and magnet. The respiration signal was transferred from the patch-type sensor embedded with a Bluetooth module to a mobile device. The signal was then displayed through the in-house Android application and stored in the device. After the training, the respiration signal can be transmitted from the mobile device to the database through the network. Equipment such as RPM, Vison-RT, and C-RAD, which use cameras to monitor markers (reflectors) on the patient’s surface or the surface movement itself, requires additional space to install the system with staff operating the system for breathing training. However, the system we developed has a tiny sensor and magnet about the size of a coin and attached to the patient’s skin, allowing them to self-train themselves. This system can dramatically reduce the workload of a hospital by enabling the patient to perform respiratory training at any position anytime and anywhere at a low cost while having similar or superior precision compared to existing respiratory training equipment.

**Figure 2:**
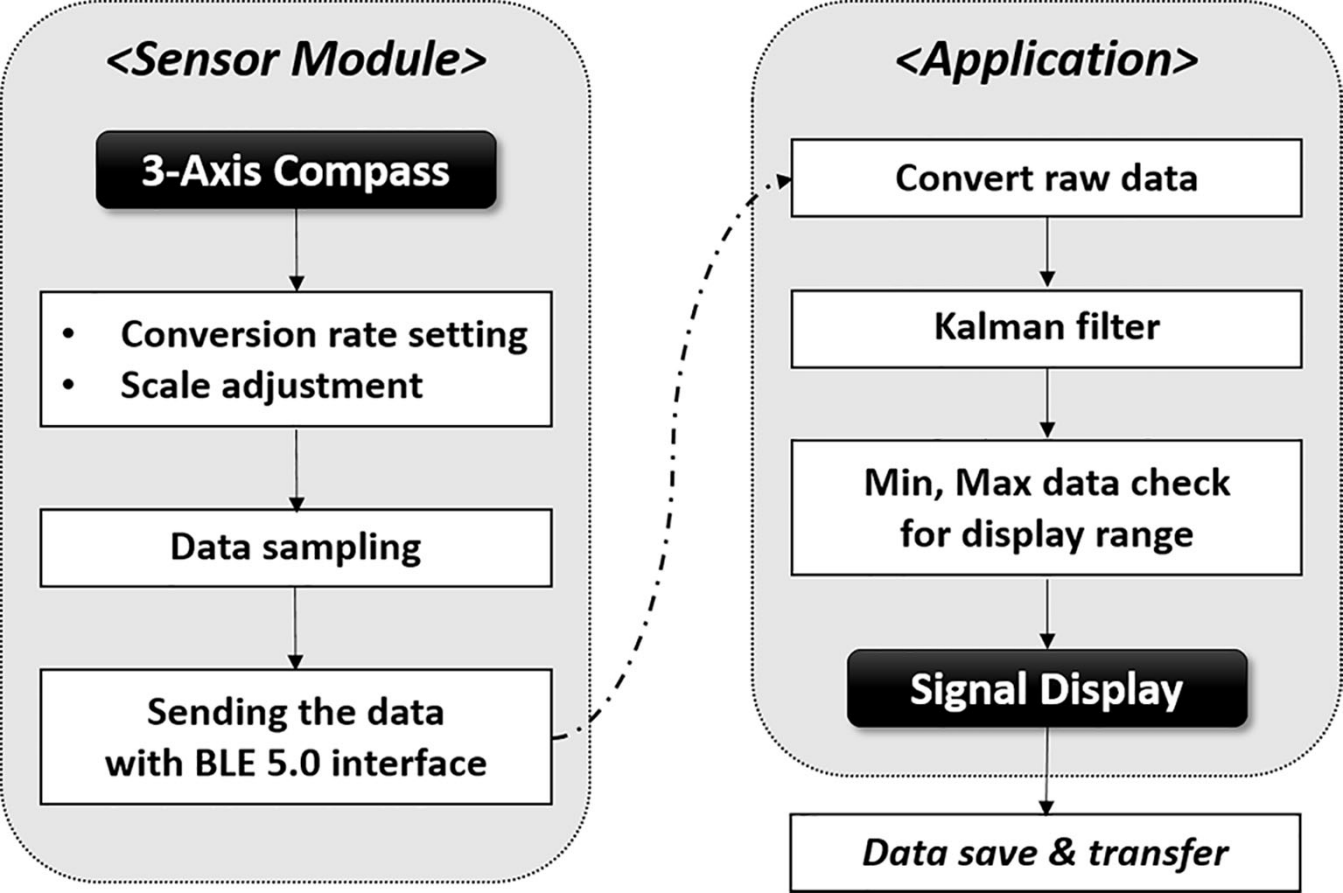
System flowchart for displaying respiratory signal in the mobile application.

The system consists of magnet, sensor, and mobile application appearing in **Figure 1B, C**, and the system flowchart for displaying respiratory signal from sensor was shown in **Figure 2**. The strength of magnet used in this study was 3200 Gauss (G), and size was 2.5 x 4.0 x 0.5 cm^3^. The developed sensor was embedded with a micro-electro-mechanical-system (MEMS)-based magnetic sensor; its size was 1.8 x 3.5 x 0.2 cm^3^; powered by a 3V coin cell battery. The magnetic intensity can be measured in three-axis, independently. Assuming that the sensor is attached to the surface of the chest lengthways, the x refers to superior-to-inferior, y refers to left-to-right and z refers anterior-posterior direction that is identical with chest movement. The measurement range was ± 12 G, sensitivity was 0.44 mG/digit, and sampling rate was about 30 Hz. The signals obtained from the MEMS sensor are sent to the phone *via* Bluetooth BLE 5.0 interface, and the transmitted signals are denoised by Kalman filter within mobile application. The three-axis signal is converted to a one-dimensional root-mean-square (RMS) signal using the following equation before being displayed.

$$\text{RMS signal}=\sqrt{\frac{{\text{x}}^{2}+{\text{y}}^{2}+{\text{z}}^{2}}{3}}$$

RMS was used to express the magnitude of resonance considering the 3-dimensional signal’s variability for time *QUASAR™ Programmable Respiratory motion phantom*.

**Figure 3:**
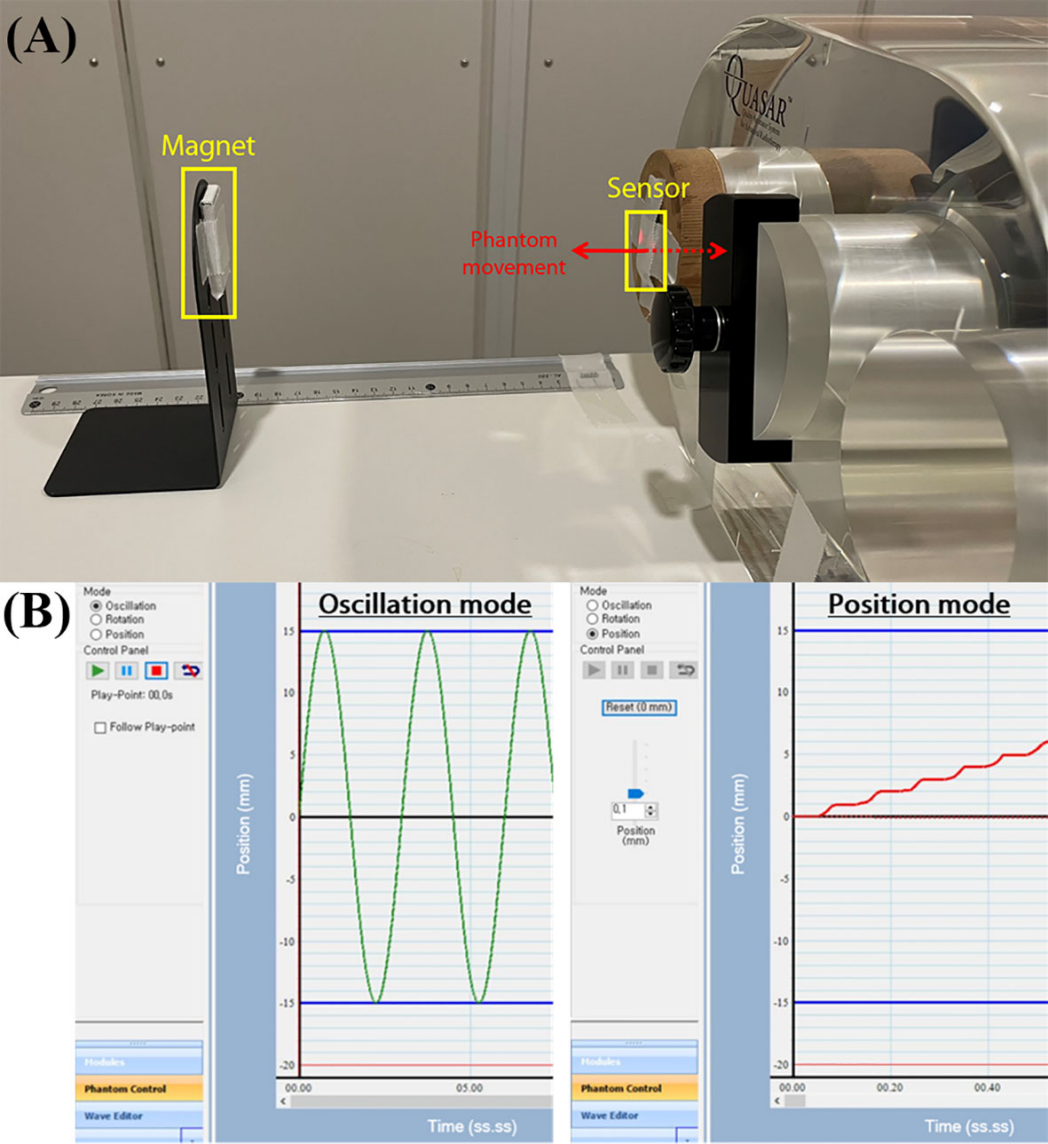
The experimental setup for sensor evaluation: **(A)** QUASAR Motion Phantom attaching the sensor to surface of the insert and the magnet aligned with sensor, **(B)** the phantom movement modes i.e., oscillation mode and position mode.

The QUASAR™ Programmable Respiratory Motion Phantom (Modus Medical Devices Inc., London, ON, Canada), can simulate various breathing patterns used to evaluate sensor performance. The respiratory motion phantom can connect to a computer controlling a motion and move in three different modes, i.e., oscillation, rotation, and position. As shown in **Figure 3**, the insert, which represents lung motion, was moved using oscillation mode and position mode with attaching the sensor to evaluate the system. The oscillation mode can allow movements in various waveforms, and the phantom provides the waveform whose amplitude range from 0.0 mm to 30.0 mm. The position mode allows up to ±20.0 mm movements in 0.1 mm increment from the reference. The oscillation mode was used to assess sensor accuracy for repetitive waveform, and the position mode was used to assess position-dependent signal change.

### Analysis Methods

Our system’s signal was transferred from a smartphone to a database using the smartphone’s wireless data communication in a text file. The text file contains the patient information, training environment information (e.g. sensor setting, patient setup, date, and time) three-axis magnetic field signal, and voltage of the battery power. The three-axis signal obtained was converted to the RMS signal for data analysis. The data obtained through QUASAR’s *oscillation mode* were analyzed in two ways as follows. The first method used the amplitude, i.e., peak-to-peak value. The difference between maximal and minimal peaks of the RMS signal was calculated for each period. The calculated average of the amplitudes was assumed identical to the amplitude set in the QUASAR, and then the error was estimated by the standard deviation of amplitudes. Additionally, the time difference between the maximal peak and the next maximal peak was calculated, whose difference from the QUASAR period was checked. The second method was to analyze the signals by fitting. In this study, the sensor was evaluated by moving the QUASAR in various periods and amplitudes of a sinewave, so the RMS signal was fit to a sine function using the following equation.

f(x)=A·sin(Bx+C)

A is the amplitude of measured data; B is the period of a sine waveform; C is the phase shift. The period from the measured signal was calculated as dividing B by 2π. Fitting was conducted using MATLAB, from which the fitting parameters were obtained to calculate the average period and amplitude for each signal; the sensor’s error was calculated by comparing it to the actual phantom motion. Data obtained through QUASAR’s *position mode* the stop signal measured at each location, so the average and standard deviation (StdDev) of the measured signal were calculated for a particular time duration for analysis. The calculated StdDev value was used as a signal-to-noise ratio (SNR) or an index of system uncertainty.

### Performance Evaluation

#### Background Signals

For accurate performance evaluation of the system, background noise was measured. The sensor was used to measure the nearby magnetic field signal, and the sensor noise was calculated as the ratio of StdDev versus the average signal. First, to confirm the background signal, i.e., the noise from the earth’s magnetic field or the ambient magnetic field, the signal was measured without placing a magnet near the sensor. Additionally, how the measurement value changed depending on the presence or absence of the magnet was confirmed. The effect of interference by the magnet residing in the phantom’s driving section was also measured. The magnet enclosed in the system was removed, and the signal was measured for analysis with the sensor placed next to the phantom in the sinewave motion. The amplitudes (P-P) of the sinewave were 10, 20, and 30 mm, and the period was 3 s.

#### Signals According to Motion Phantom’s Period and Amplitude

To evaluate the sensor’s response to various breathing patterns, the signal was measured as the motion phantom’s amplitude and period were varied. The signal was measured after attaching the sensor to the phantom’s inserting area, which shows thoracic changes due to a patient’s breathing, and placing the magnet 15 cm away from the sensor. The phantom was in sinewave motions whose amplitudes (P-P) were 10, 20, and 30 mm, and periods were 1, 2, 3, 4, and 5 s. The measurement was made twice for 15 sets, and each signal was analyzed by calculating its amplitude error and time error using the two aforementioned methods: analytic methods using amplitude value or fitting parameter.

#### Signals According to the Distance Between Sensor and Magnet

Because the distance between sensor and magnet can influence the signal, signal accuracy according to distance change was confirmed. The signal was measured twice for the distance between sensor and magnet at 10, 15, 20, and 25 cm when the phantom was moved in sinewave of 10 mm amplitude (P-P) and 3 s periods. To calculate the accuracy, each signal was analyzed using the two aforementioned methods: analytic methods using amplitude value or fitting parameter.

#### Signals According to Phantom’s Position Change

Using the QUASAR’s *position mode*, we evaluated the sensor’s sensitivity to the phantom’s position change in the stationary state. The distance between sensor and magnet was 16 cm. With this as a reference, the signal was measured as the phantom position was moved in 1 mm increments between -10 to 10 mm. For the stop section after the position change, the average signal and StdDev were calculated, and the lengths for the corresponding segments were identically analyzed. An identical experiment was performed three times, and the position accuracy was calculated with the phantom motion as reference.

#### Signals According to the Absence and Presence of Material Between the Sensor and Magnet

**Figure 4:**
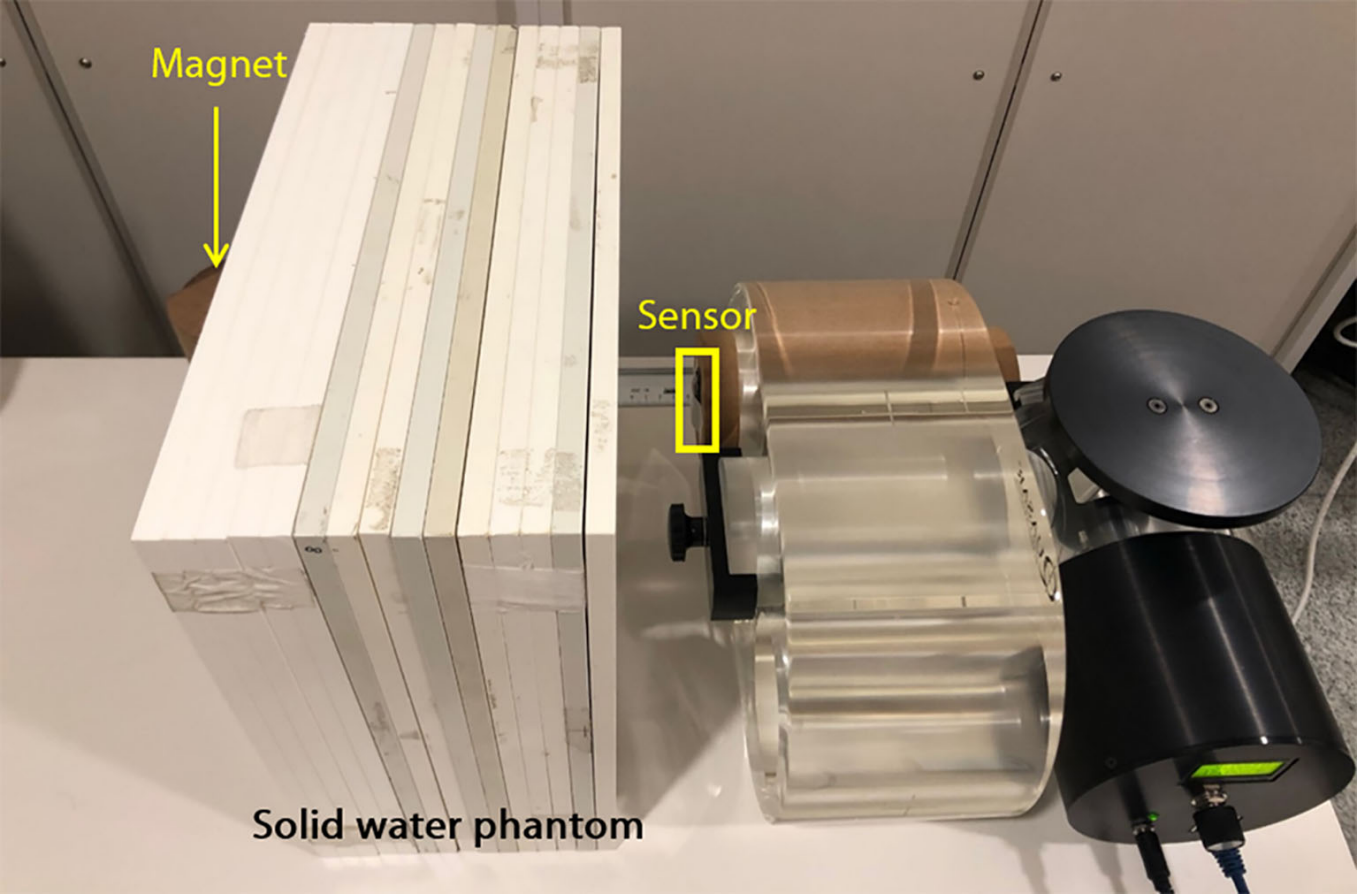
The experimental setup for evaluation of signals according to the absence and presence of material between the sensor and magnet.

The developed respiratory training system operates *via* a sensor attached to the chest and a magnet placed on the back to monitor respiration, so a patient’s body lies between the sensor and the magnet. The change in the signal caused by the material between the sensor and the magnet was evaluated. As shown in **Figure 4**, the sensor was attached to the phantom, and the magnet was placed 20 cm away with a solid water phantom, e.g. slab phantom (RW3, PTW Freiburg), placed in between. The thickness of the slab phantom was 15 cm. Air was in between when there was no slab phantom. The phantom was moved in a sinewave of 3 s periods and 10-, 20-, and 30-mm amplitudes. Each signal was analyzed using the two methods previously mentioned, and the total error was calculated as the average of the errors obtained using the methods individually.

## Results

### Background Signals

**Figure 5:**
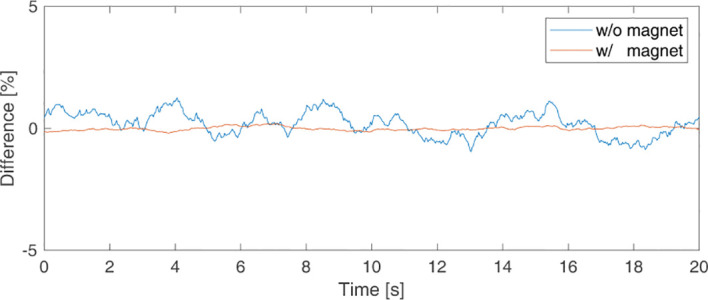
Background signal picked up by the sensor to check for sensor noise when there was (in orange line) and was not (in blue line) a magnet.

**Figure 5** shows a signal measured without a magnet and a signal measured with magnet placed 15 cm from the sensor. The difference [%] between the averaged signal and the measured signal over time is shown. The magnitude of the measured signal without a magnet was 1981.4 ± 10.073, and the signal-to-ratio (SNR), the ratio of StdDev versus the average signal, was 0.54%. The magnitude of the signal measured with a magnet 15cm away from the sensor was 18714.8 ± 15.082, and the SNR was 0.08%. Additionally, to confirm the effect of the magnet inside the phantom on the measurement, the signal was measured with the sensor placed next to the phantom moving in sinusoidal form. **Table 1** shows the mean and StdDev of the signals according to the amplitude of sinewave. The greater the moving amplitude of the phantom, the greater the StdDev of the signal.

**Table 1:** Average and standard deviation of signals according to the amplitude of the motion phantom by magnets inside the phantom.

Amplitude (P-P)	10 mm	20 mm	30 mm
**Average signal**	3106.25	3087.93	3084.24
**StdDev**	20.90	35.736	50.785

### Signals According to Motion Phantom’s Period and Amplitude

**Figure 6:**
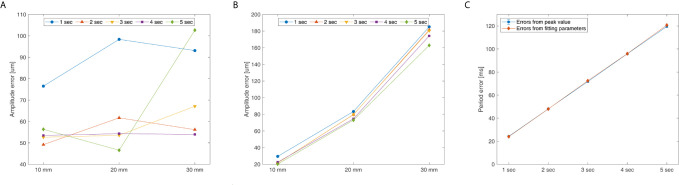
System’s amplitude and period error for the QUASAR Motion Phantom’s amplitude (P-P) and cycle: **(A)** Peak value analyzing amplitude error, **(B)** Fitting parameter analyzing amplitude error, and **(C)** Peak value and fitting parameter analyzing period error.

**Figure 6** shows the measurement accuracy according to the motion phantom’s period and amplitude, and **Figure 6A** shows the results from analyzing the measured amplitude signal using the peak value. Regarding the measurements of the amplitude precision, the amplitude error of the signal was the smallest (46.57 μm) at an amplitude of 20 mm and a period of 5 s, and was the largest (102.65 μm) at an amplitude of 30 mm and a period of 5 s. **Figure 6B** shows the results from analyzing the measured signal using the fitting parameter. The amplitude error was the smallest (20.27 μm) at an amplitude of 10 mm and a period of 5 s, and was the largest (185.15 μm) at an amplitude of 30 mm and a period of 1 s. **Figure 6C** shows the periodic precision of the measured signal analyzed by using the peak value and fitting parameter. For both methods, the periodic error increased as the motion phantom’s period increased, and the difference in periodic error between the two methods was <2 ms. The period error was the largest at 121 ms when the period was 5 s.

### Signals According to the Distance Between Sensor and Magnet

**Figure 7:**
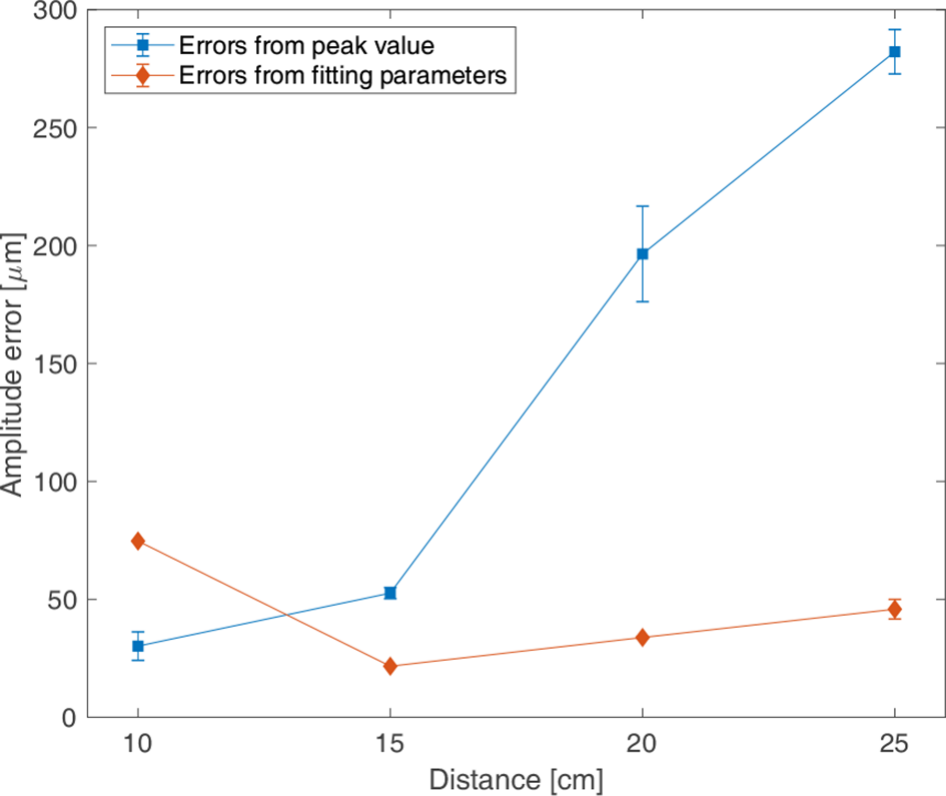
Amplitude errors for the distance between the sensor and magnet according to the motion phantom moving in sinewave of 3 s periods and of 10 mm amplitude (P-P); the errors from peak values (in blue and ■), the errors from fitting parameter (in pink and ◆).

**Figure 7** shows the precision of the measured signal according to the change in distance between the sensor attached to the motion phantom and static magnet. The errors calculated by peak value method and fitting parameter method were shown according to the distance. Measurements were made twice for each distance, and the error calculated in each measurement were averaged. The x-axis is the distance between the sensor and the static magnet, and the y-axis is the amplitude error calculated from the peak value method and from the fitting parameter method. When precision was evaluated using the peak value, the error was the minimum at 30.17 μm at a 10 cm distance, and it was the maximum at 196.43 μm at 20 cm. When precision was evaluated using a fitting parameter, the error was the minimum at 21.65 μm at 15 cm, and it was the maximum at 74.70 μm at 10 cm. Periodic errors calculated using the peak value and fitting parameter were 71.34 ms and 72.21 ms, respectively; the difference in the periodic errors according to distance was <2 ms.

### Signals According to Phantom’s Position Change

**Figure 8:**
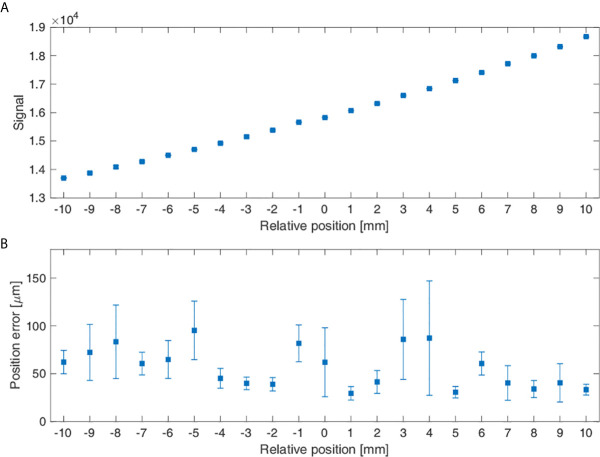
Measurement signal reflecting the phantom position change: **(A)** Average signal according to the sensor’s relative positions and **(B)** The sensor’s position errors according to its relative position.

**Figure 8** shows the average signal measured for the stationary state when the phantom position was displaced by 1 mm increments and its errors. In **Figure 8A**, when the phantom’s relative position was varied from -10 to 10 mm, the average value of the signal increased with the increase in the relative position value. **Figure 8B** shows the position errors at each location, which had a maximum of 95.28 μm at -5 mm and a minimum of 29.50 μm at 1 mm.

### Signals According to the Absence and Presence of Material Between the Sensor and Magnet

**Figure 9:**
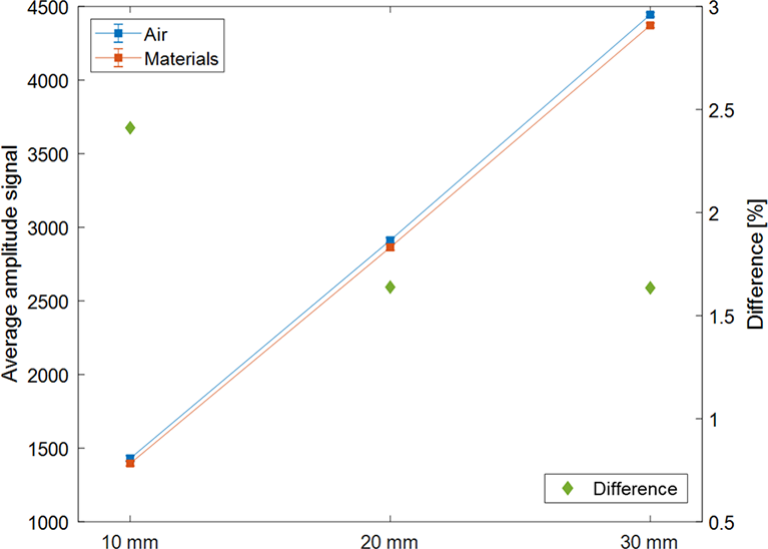
Average measurement signal according to the type of materials between the sensor and magnet (air in blue; solid water phantom in orange) and the difference between the two average signals (in green and ◆) expressed according to the amplitude (P-P) of the motion phantom.

**Figure 9** shows the mean value and the error of measured signal with and without solid water phantom between magnet and sensor, and the difference between the two signals was shown according to the amplitude of the motion phantom. The signal differences according to the absence and presence of the phantom between the magnet and sensor were 2.41%, 1.64%, and 1.63% for 10, 20, and 30 mm amplitudes.

## Discussion

The respiratory training system we developed utilizes a wearable patch-type sensor and a personal mobile device, enable for patients to conduct respiratory training themselves without any restriction for time and space. The present study evaluated the basic performance of the sensor system and its clinical usability.

The sensor’s noise level was measured to be 0.54% of the signal and 0.08% in the absence and presence of strong magnetic fields, respectively. Additionally, in the analysis of the magnet’s influence—existing in the motor inside the QUASAR phantom—on the signal, the signal change due to the QUASAR’s movement was 0.673–1.643% of the signal. Dunn L et al. reported that a new generation of QUASAR™ motion phantom accuracy is about 0.1 mm (13). The measurement errors included the error of the sensor itself and the error of the motion itself implemented by the motion phantom. Therefore, it is not significantly different from the uncertainty caused by the motion phantom.

The monitoring accuracy of the system for amplitude and period changes in the motion phantom with sinewave motions were a mean amplitude and period errors of 78.87 um and 72 ms, respectively. The up and down motion of the patient’s chest surface due to respiration is approximately 1 cm (14). Regarding the amplitude monitoring accuracy, supposing respiration cycle is divided into ten phases in four-dimensional computed tomography (4DCT), our system was analyzed to have a <10% resolution of the moving position per phase (about 1 mm), which is considered to be clinically applicable. With respect to the accuracy of period monitoring, assuming a patient’s respiratory cycle is about 3 s (15), the system was analyzed to have an accuracy of within 4%. The sensor error calculated based on the measured data contains the position and time errors intrinsic to the QUASAR motion phantom. According to the vendor-provided information, the position and time accuracy of the phantom is 0.1 mm and 10 ms, respectively, which is comparable to the system error obtained in this experiment.

The AAPM TG report 142 recommends for the temporal accuracy of a phase <100 ms as tolerance for the respiratory gating system’s annual quality assurance (QA) procedure (16). Through the performance evaluation using Varian’s Respiratory Gating for Scanners (RGSC; Varian Medical Systems, Palo Alto, CA, USA) by Chengyu Shi, et al., the system’s amplitude accuracy was recommended to be <2 mm and phase accuracy to be <100 ms in periodic QA (17). Additionally, Fattorl et al. reported amplitude and period discrepancies of 400 μm and 12.71 ms, respectively, from evaluating a real-time optical tracking system using six markers (18). Compared to the precision of commercial respiratory monitoring systems, our MEMS-based system could be considered to have clinically relevant precision.

Apart from the accuracy of respiratory movements, the measurement of position accuracy in a stationary state showed that the position error was a minimum of 29.50 μm and a maximum of 95.28 μm for 1 mm displacement. Recently, the breath-hold method was introduced in which a sleep apnea therapy device, such as continuous positive airway pressure (CPAP), is used to reduce irradiation on the heart and the lungs by increasing pulmonary volume and the distance between the heart and the planning target volume (PTV) (19). In this context, the capability for precise localization can demonstrate the clinical efficacy of the system that allows individuals to conduct breath-hold respiratory training themselves.

The measurements were conducted to confirm the effect of the distance between the magnet and sensor on the signals. The error was the smallest for a distance of 15 cm, and the stable signals can be obtained when the distance is 10-25 cm, which is the thickness of the patient’s body. This system, which monitors respiration by attaching a sensor to the patient’s chest and magnet to the back, could be applied for patients whose body thickness is over 30 cm by using a high strength magnet. In this case, it is necessary to evaluate the sensor’s response to magnets with different strengths before clinical application.

The experiments to confirm the effect of the presence or absence of material between the sensor and the magnet on the signal were conducted. The difference of the average signal was 1.89%, indicating that the difference is not substantial. Therefore, if the material does not get magnetized between the sensor and the magnet, its presence or absence would not affect the respiratory signal, but the distance between the sensor and magnet is essential.

Using this system, the patients can monitor their respiration and do respiratory training anytime, anywhere because the system does not require a separate space and is easy to control. The demonstration video of healthy volunteer using the system to monitor respiration was founded in the **Supplementary Material**. In the following study, the system will be upgraded to provide a guide for regular breathing or to provide a reference point for deep inspiration breath-hold (DIBH) training.

## Conclusion

In this study, the respiratory self-training system was developed using a patch-type magnetic sensor with wireless communication capabilities and evaluated its basic performance and clinical applicability using the QUASAR™ Respiratory Motion Phantom. The system is wearable, convenient to use, and patients could use it without space and time constraints to utilize respiratory monitoring and training with a suitable level of precision. Our findings suggest that using the wearable respiratory self-training system for radiation therapy could be considered to provide precise respiratory gating. If the clinical efficacy of the system is evaluated in future clinical trials, this system will benefit many patients in need of respiratory training.

## Data Availability Statement

The raw data supporting the conclusions of this article will be made available by the authors, without undue reservation.

## Author Contributions

Conception, design, and drafting the manuscript were performed by HKK, JSK, and DK. Data acquisition, analysis, and interpretation were performed by HKK, HJK, C-SH, JHK, JSK, and DK. All authors contributed to the article and approved the submitted version.

## Funding

This work was supported by a faculty research grant of Yonsei University College of Medicine (6-2019-0104), the general researcher program (NRF- 2018R1D1A1B07050217) and the nuclear safety research program (No. 2003013) through the Korea Foundation of Nuclear Safety (KOFONS), using the financial resource granted by the Nuclear Safety and Security Commission (NSSC), Republic of Korea.

## Conflict of Interest

The authors declare that the research was conducted in the absence of any commercial or financial relationships that could be construed as a potential conflict of interest.

## Publisher’s Note

All claims expressed in this article are solely those of the authors and do not necessarily represent those of their affiliated organizations, or those of the publisher, the editors and the reviewers. Any product that may be evaluated in this article, or claim that may be made by its manufacturer, is not guaranteed or endorsed by the publisher.

## Abbreviations

3D-CRT, three-dimensional conformal radiation therapy; IMRT, intensity-modulated radiation therapy; VMAT, volumetric modulated arc therapy; SBRT, stereotactic body radiation therapy; OARs, organ at risks; ITV, internal target volume; CTV, clinical target volume; PTV, planning target volume; MEMS, micro-electric-mechanical-system; RMS, root-mean-square; StdDev, standard deviation; SNR, signal-to-noise ratio; P-P, peak-to-peak; 4DCT, four-dimensional computed tomography; CPAP, continuous positive airway pressure.

## References

[1] Wambersie A. ICRU Report 62, Prescribing, Recording and Reporting Photon Beam Therapy (Supplement to ICRU Report 50). ICRU News (1999).

[2] Mutaf YD Brinkmann DH. Optimization of Internal Margin to Account for Dosimetric Effects of Respiratory Motion. Int J Radiat Oncol Biol Phys (2008) 70(5):1561–70. 10.1016/j.ijrobp.2007.12.025 18374230

[3] Kwa SL Lebesque JV Theuws JC Marks LB Munley MT Bentel G. Radiation Pneumonitis as a Function of Mean Lung Dose: An Analysis of Pooled Data of 540 Patients. Int J Radiat Oncol Biol Phys (1998) 42(1):1–9. 10.1016/S0360-3016(98)00196-5 9747813

[4] Hope AJ Lindsay PE El Naqa I Alaly JR Vicic M Bradley JD. Modeling Radiation Pneumonitis Risk With Clinical, Dosimetric, and Spatial Parameters. Int J Radiat Oncol Biol Phys (2006) 65(1):112–24. 10.1016/j.ijrobp.2005.11.046 16618575

[5] Pollock S Keall R Keall P. Breathing Guidance in Radiation Oncology and Radiology: A Systematic Review of Patient and Healthy Volunteer Studies. Med Phys (2015) 42(9):5490–509. 10.1118/1.4928488 26328997

[6] Yoganathan SA Maria Das KJ Agarwal A Kumar S. Magnitude, Impact, and Management of Respiration-Induced Target Motion in Radiotherapy Treatment: A Comprehensive Review. J Med Phys (2017) 42(3):101–15. 10.1016/j.ijrobp.2013.04.012 PMC5618455 28974854

[7] Mori S Knopf AC Umegaki K. Motion Management in Particle Therapy. Med Phys (2018) 45(11):e994–e1010. 10.1002/mp.12679 30421815

[8] Keall PJ Mageras GS Balter JM Emery RS Forster KM Jiang SB. The Management of Respiratory Motion in Radiation Oncology Report of AAPM Task Group 76 a. Med Phys (2006) 33(10):3874–900. 10.1118/1.2349696 17089851

[9] Giraud P Houle A. Respiratory Gating for Radiotherapy: Main Technical Aspects and Clinical Benefits. ISRN Pulmonol (2013) 2013:1–13. 10.1155/2013/519602 20605765

[10] Venkat RB Sawant A Suh Y George R Keall PJ. Development and Preliminary Evaluation of a Prototype Audiovisual Biofeedback Device Incorporating a Patient-Specific Guiding Waveform. Phys Med Biol (2008) 53(11):N197–208. 10.1088/0031-9155/53/11/N01 18475007

[11] Kini VR Vedam SS Keall PJ Patil S Chen C Mohan R. Patient Training in Respiratory-Gated Radiotherapy. Med Dosimetry (2003) 28(1):7–11. 10.1016/S0958-3947(02)00136-X 12747612

[12] Oh Y Jung YJ Choi SH Kim DW. Design and Evaluation of a MEMS Magnetic Field Sensor-Based Respiratory Monitoring and Training System for Radiotherapy. Sensors (Basel) (2018) 18(9):1–10. 10.3390/s18092742 PMC6163714 30134526

[13] Dunn L Kron T Johnston P McDermott L Taylor M Callahan J. A Programmable Motion Phantom for Quality Assurance of Motion Management in Radiotherapy. Australas Phys Eng Sci Med (2012) 35(1):93–100. 10.1007/s13246-011-0114-0 22119931

[14] Ahn S Yi B Suh Y Kim J Lee S Shin S. A Feasibility Study on the Prediction of Tumour Location in the Lung From Skin Motion. Brit J Radiol (2004) 77(919):588–96. 10.1259/bjr/64800801 15238406

[15] Lovett PB Buchwald JM Stürmann K Bijur P. The Vexatious Vital: Neither Clinical Measurements by Nurses Nor an Electronic Monitor Provides Accurate Measurements of Respiratory Rate in Triage. Ann Emerg Med (2005) 45(1):68–76. 10.1016/j.annemergmed.2004.06.016 15635313

[16] Klein EE Hanley J Bayouth J Yin FF Simon W Dresser S. Task Group 142 Report: Quality Assurance of Medical Accelerators. Med Phys (2009) 36(9):4197–212. 10.1118/1.3190392 19810494

[17] Shi C Tang X Chan M. Evaluation of the New Respiratory Gating System. Precis Radiat Oncol (2017) 1(4):127–33. 10.1002/pro6.34 PMC5927385 29722356

[18] Fattori G Hrbacek J Regele H Bula C Mayor A Danuser S. Commissioning and Quality Assurance of a Novel Solution for Respiratory-Gated PBS Proton Therapy Based on Optical Tracking of Surface Markers. Z Med Phys (2020). 10.1016/j.zemedi.2020.07.001 PMC9948868 32830006

[19] Kil WJ Pham T Hossain S Casaigne J Jones K Khalil M. The Impact of Continuous Positive Airway Pressure on Radiation Dose to Heart and Lung During Left-Sided Postmastectomy Radiotherapy When Deep Inspiration Breath Hold Technique Is Not Applicable: A Case Report. Radiat Oncol J (2018) 36(1):79–84. 10.3857/roj.2018.00017 29506325 PMC5903364

