# Supplementary material for: Development and Performance Evaluation of Wearable Respiratory Self-Training System Using Patch Type Magnetic Sensor
Source: Front Oncol. 2021 Aug 3;11:680147. doi: 10.3389/fonc.2021.680147 (PMC8370089; doi:10.3389/fonc.2021.680147)
Supplement: Supplementary file 1 [file Table_1.docx]

Supplementary Material

# Supplementary Data

Table S1. Amplitude error and period error that are analyzed by peak-to-peak method and fitting method according to amplitude and period of QUASAR motion phantom moving in a sinusoidal wave.

|  | Amplitude Error [$\boldsymbol{\mu}$m] | | Period Error [ms] | | |
| --- | --- | --- | --- | --- | --- |
| Analysis  method | Peak-to-peak value method | Fitting method | | Peak-to-peak value method | Fitting method |
| Amplitude: 10 mm |  |  | |  |  |
| Period |  |  | |  |  |
| 1 s | 76.51 | 29.50 | | 0.025 | 0.024 |
| 2 s | 49.17 | 21.62 | | 0.048 | 0.048 |
| 3 s | 52.64 | 21.65 | | 0.071 | 0.072 |
| 4 s | 53.49 | 22.55 | | 0.095 | 0.096 |
| 5 s | 56.35 | 20.27 | | 0.119 | 0.121 |
| Amplitude: 20 mm |  |  | |  |  |
| Period |  |  | |  |  |
| 1 s | 98.36 | 83.40 | | 0.024 | 0.024 |
| 2 s | 61.70 | 79.65 | | 0.048 | 0.048 |
| 3 s | 53.54 | 79.86 | | 0.072 | 0.072 |
| 4 s | 54.31 | 74.65 | | 0.097 | 0.096 |
| 5 s | 46.57 | 73.05 | | 0.120 | 0.121 |
| Amplitude: 30mm |  |  | |  |  |
| Period |  |  | |  |  |
| 1 s | 93.12 | 185.15 | | 0.024 | 0.024 |
| 2 s | 56.17 | 181.46 | | 0.048 | 0.048 |
| 3 s | 67.12 | 180.49 | | 0.072 | 0.072 |
| 4 s | 53.96 | 174.23 | | 0.096 | 0.096 |
| 5 s | 102.65 | 162.88 | | 0.120 | 0.121 |

Table S2. Amplitude error and period error that are analyzed by peak-to-peak method and fitting method according to distance between sensor and magnet; QUASAR^TM^ motion phantom was moving in a sinusoidal wave that was amplitude of 10 mm and period of 3 seconds.

|  | Amplitude Error [$\boldsymbol{\mu}$m] | | Period Error [ms] | | |
| --- | --- | --- | --- | --- | --- |
| Analysis  method | Peak-to-peak value method | Fitting method | | Peak-to-peak value method | Fitting method |
| Distance [cm] |  |  | |  |  |
| 10 | 30.17 | 74.70 | | 72.30 | 72.46 |
| 15 | 52.64 | 21.65 | | 71.48 | 72.46 |
| 20 | 196.43 | 33.82 | | 70.24 | 71.71 |
| 25 | 282.16 | 45.82 | | 73.13 | 72.50 |
